# Canonical NF-κB Promotes Lung Epithelial Cell Tumour Growth by Downregulating the Metastasis Suppressor CD82 and Enhancing Epithelial-to-Mesenchymal Cell Transition

**DOI:** 10.3390/cancers13174302

**Published:** 2021-08-26

**Authors:** Eugenia Roupakia, Evangelia Chavdoula, Georgia Karpathiou, Giannis Vatsellas, Dimitrios Chatzopoulos, Angeliki Mela, Jennifer M. Gillette, Katharina Kriegsmann, Mark Kriegsmann, Anna Batistatou, Anna Goussia, Kenneth B. Marcu, Emmanouil Karteris, Apostolos Klinakis, Evangelos Kolettas

**Affiliations:** 1 Laboratory of Biology, School of Medicine, Faculty of Health Sciences, Institute of Biosciences, University Research Centre, University of Ioannina, University Campus, 45110 Ioannina, Greece; ev.roupakia@gmail.com; 2 Biomedical Research Division, Institute of Molecular Biology and Biotechnology, Foundation for Research and Technology, University of Ioannina Campus, 45115 Ioannina, Greece; echavdoula@gmail.com; 3 Biomedical Research Foundation, Academy of Athens (BRFAA), 4 Soranou Ephessiou Street, 11527 Athens, Greece; gvatsellas@bioacademy.gr (G.V.); dimitrischat@gmail.com (D.C.); kenneth.marcu@stonybrook.edu (K.B.M.); aklinakis@bioacademy.gr (A.K.); 4 Laboratory of Pathology, School of Medicine, Faculty of Health Sciences, University of Ioannina, 45500 Ioannina, Greece; gakarpath@yahoo.gr (G.K.); abatista@uoi.gr (A.B.); agoussia@uoi.gr (A.G.); 5 Department of Pathology and Cell Biology Columbia University Medical Center, Irving Comprehensive Cancer Research Center, Columbia University, New York, NY 10032, USA; am2904@cumc.columbia.edu; 6 Department of Pathology, School of Medicine, University of New Mexico, Albuquerque, NM 87131, USA; jgillette@salud.unm.edu; 7 Department of Internal Medicine V, University Hospital Heidelberg, 69120 Heidelberg, Germany; Katharina.Kriegsmann@med.uni-heidelberg.de; 8 Institute of Pathology, University Hospital Heidelberg, 69120 Heidelberg, Germany; Mark.Kriegsmann@med.uni-heidelberg.de; 9 Department of Biochemistry and Cell Biology, Microbiology and Pathology, Stony Brook University, New York, NY 11794, USA; 10 Division of Biosciences, Department of Life Sciences, College of Health, Medicine and Life Sciences, Brunel University London, Uxbridge, Middlesex, London UB8 PH, UK; emmanouil.karteris@brunel.ac.uk

**Keywords:** human NSCLC models, NF-κB RelA/p65, RNA-seq, CD82, cell migration, EMT, integrin signalling

## Abstract

**Simple Summary:**

Canonical NF-κB signalling pathway acts as a tumour promoter in several types of cancer including non-small cell lung cancer (NSCLC), but the mechanism(s) by which it contributes to NSCLC is still under investigation. We show here that NF-κB RelA/p65 is required for the tumour growth of human NSCLC cells grown in vivo as xenografts in immune-compromised mice. RNA-seq transcriptome profile analysis identified the metastasis suppressor *CD82/KAI1*/*TSPAN27* as a canonical NF-κB target. Loss of CD82 correlated with malignancy. RelA/p65 stimulates cell migration and epithelial-to-mesenchymal cell transition (EMT), mediated, in part, by CD82/KAI1, through integrin-mediated signalling, thus, identifying a mechanism mediating NF-κB RelA/p65 lung tumour promoting function.

**Abstract:**

Background: The development of non-small cell lung cancer (NSCLC) involves the progressive accumulation of genetic and epigenetic changes. These include somatic oncogenic *KRAS* and *EGFR* mutations and inactivating *TP53* tumour suppressor mutations, leading to activation of canonical NF-κB. However, the mechanism(s) by which canonical NF-κB contributes to NSCLC is still under investigation. Methods: Human NSCLC cells were used to knock-down RelA/p65 (RelA/p65^KD^) and investigate its impact on cell growth, and its mechanism of action by employing RNA-seq analysis, qPCR, immunoblotting, immunohistochemistry, immunofluorescence and functional assays. Results: RelA/p65^KD^ reduced the proliferation and tumour growth of human NSCLC cells grown in vivo as xenografts in immune-compromised mice. RNA-seq analysis identified canonical NF-κB targets mediating its tumour promoting function. RelA/p65^KD^ resulted in the upregulation of the metastasis suppressor *CD82/KAI1*/*TSPAN27* and downregulation of the proto-oncogene *ROS1*, and *LGR6* involved in Wnt/β-catenin signalling. Immunohistochemical and bioinformatics analysis of human NSCLC samples showed that CD82 loss correlated with malignancy. RelA/p65^KD^ suppressed cell migration and epithelial-to-mesenchymal cell transition (EMT), mediated, in part, by CD82/KAI1, through integrin-mediated signalling involving the mitogenic ERK, Akt1 and Rac1 proteins. Conclusions: Canonical NF-κB signalling promotes NSCLC, in part, by downregulating the metastasis suppressor CD82/KAI1 which inhibits cell migration, EMT and tumour growth.

## 1. Introduction

Non-small cell lung cancer (NSCLC), one of the most common cancers worldwide, with high incidence and mortality rates, is histologically divided into three major subtypes: adenocarcinoma (LUAD) (~70%), squamous cell carcinoma (LUSC) (~20%), and large cell lung carcinoma (~10%). The development of these histological subtypes occurs through the progressive accumulation of genetic and epigenetic events, with inactivating mutations in the p53 tumour suppressor detected in >50% of the cases, being common to all subtypes [1,2,3,4,5,6,7,8,9]. The most commonly mutated genes in LUAD include *KRAS* and *EGFR*, and at a lower frequency *BRAF*, *ALK*, *PIK3CA* and *ROS1* oncogenes, and in the tumour suppressor genes *LKB1* (STK11), *NF1* and *PTEN* [3,4,6,8,9]. Murine cancer models have been used to evaluate the impact of the genetic changes in LUAD onset, development and progression [10]. Importantly, several studies have revealed a link between oncogenic K-Ras [11,12,13,14] or EGFR proteins [15,16,17] and increased canonical NF-κB activity in NSCLC [7].

NF-κB transcription factors (TFs) are critical regulators of pro-inflammatory and stress-like responses. NF-κB TFs bind to DNA as heterodimers or homodimers composed of 5 subunits: RelA/p65, c-Rel, RelB, p50, p52. All NF-κB family members contain an N-terminal DNA binding and dimerisation domain known as the Rel homology domain. The c-Rel (*C-REL*), p65/RelA (*RELA*) and RelB (*RELB*) subunits contain a C-terminal transactivation domain, but p50 and p52, which are derived by processing of the larger precursors p50/p105 (NF-κB1) and p52/p100 (NF-κB2), respectively, lack a transcriptional activation domain. Activation of NF-κB is achieved by two main signalling pathways: An IKKβ-mediated canonical NF-κB pathway and an IKKα-mediated non-canonical or alternative NF-κB pathway. In the former, c-Rel/p50 and RelA(p65)/p50 heterodimers are restrained in the cytoplasm of most cells not experiencing a pro-inflammatory/stress-like response by NF-κB inhibitors, the IκBs bound to them. Activation of canonical NF-κB pathway is initiated by the phosphorylation of serines (Ser) 32 and 36 of IκBα causing its ubiquitination and proteasomal degradation, resulting in c-Rel/p50 or p65/50 heterodimer nuclear translocation to regulate target gene expression. Phosphorylation of IκBα at Ser32/36 is mediated by the IKK signalsome complex composed of the upstream activating IKKα and IKKβ Ser/Thr kinases, and NEMO/IKKγ, a regulatory/adaptor protein. Activation of IKKβ by Ser177/181 phosphorylation is NEMO-dependent and occurs in response to pro-inflammatory and stress signals. Activation of the non-canonical or alternative NF-κB pathway involves the NF-κB Inducing Kinase (NIK)-dependent phosphorylation of IKKα at Ser176/180. Activated IKKα then mediates the phosphorylation of p100/NF-κB2, inducing its ubiquitination and proteasome-dependent processing yielding the mature p52 subunit and p52/RelB heterodimers that translocate to the nucleus and regulate non-canonical NF-κB target genes [18,19,20,21].

The NF-κB TFs can either activate or repress target gene transcription in different physiological contexts [19,22,23]. NF-κB-induced genes regulate cancer cell proliferation, survival, metastasis and angiogenesis [18,19,20]. NF-κB TFs function as tumour promoters within transformed cells, but also influence the host’s innate immune response to cancer cells by regulating functions of infiltrating lymphocytes and macrophages and promoting inflammation in the tumour microenvironment [21].

The NF-κB TFs and their immediate upstream signalling components are aberrantly expressed and/or activated in inflammatory lung diseases and NSCLC and have been implicated in the unfavourable prognosis of patient survival [7,24]. Several studies including murine models have revealed a link between oncogenic *KRAS* [11,12,13,14] and *EGFR* [15,16,17] and increased canonical NF-κB activity in mouse lung cancer models and human lung epithelial cells, and the induction of an inflammatory response in lung tumours. Similarly, murine models have uncovered functional effects of IKKβ and of the canonical NF-κB RelA/p65 subunit in urethane- induced inflammation and NSCLC [25,26]. In both the urethane- and the oncogene-induced mouse lung cancer models suppression of canonical NF-κB using an IκBαSR super-repressor [11,25] or by ablation of RelA/p65 [12] lead to impairment of tumour growth and spread in conjunction with the accumulation of apoptotic cells [12]. Similarly, ablation of IKKβ in lung epithelium [13] or in myeloid cells [14] reduced K-Ras^G12D^-induced inflammation, proliferation and tumour growth in mice. Ablation of IKKβ in alveolar type (AT)-II lung cells also reduced urethane-induced LUAD [26]. Mouse strains susceptible to urethane-induced lung carcinogenesis exhibit NF-κB activation and lung inflammation, in the airway and AT-II lung cells and macrophages suggesting that canonical NF-κB signalling in airway epithelium is required for urethane-induced lung carcinogenesis [25].

However, despite the aforementioned studies on the requirement and the tumour promoting role of canonical NF-κB in human and mouse NSCLC, the underlying mechanisms are still under investigation. By performing an integrative cytogenetic and gene expression analysis of NSCLC and SCLC cell lines and tumours, a recurring amplification at chromosome 11p13 was identified, that only contained TNF receptor-associated factor 6 (TRAF6). Inhibition of TRAF6 in human lung cancer cells suppressed NF-κB activation, anchorage-independent and tumour growth. Thus, TRAF6 acted as an oncogene leading to the constitutive NF-κB activation in K-Ras-driven lung cancers [27]. Ablation of IKKβ in K-Ras^G12D^-induced LUAD in mice resulted in decreased tumour growth and burden due to reduced AT-II lung cell proliferation. This was due to the down-regulation of several E2F target genes implicated in cell cycle progression, the mitotic checkpoint Ser/Thr kinase BUB1, and to Timp1 (tissue inhibitor of metalloproteinase 1) which was identified as a canonical NF-κB target gene involved in lung tumourigenesis [13]. Activation of canonical NF-κB in response to EGFR oncogene inhibition in mutant EGFR-bearing human and mouse lung cancer cells, drives tumour cell survival and residual disease in lung cancer via several established regulators of canonical NF-κB signalling and cell survival such as TNFAIP3 (TNFα-induced protein 3), BIRC3 (c-IAP2, TNFR2-TRAF signalling complex protein) and IL-6 [16].

In the present study, we show that RelA/p65 functions as an epithelial tumour promoter in human NSCLC cell lines (harbouring either wild-type or mutant *KRAS* and *TP53* genes) grown as tumour xenografts in immune-compromised NSG mice. We identified the metastasis suppressor CD82 as a RelA/p65 target. CD82 expression was downregulated in LUAD and LUSC. We also provide evidence that RelA/p65′s tumour promoting action in NSCLC is due to, at least in part, the downregulation of CD82 that inhibits cell migration and EMT, and the stimulation of ERK and Rac1 through the engagement of integrin-mediated signalling as revealed by bioinformatics analysis.

## 2. Results

### 2.1. RelA/p65 Was Required for Tumour Growth In Vivo

**Figure 1:**
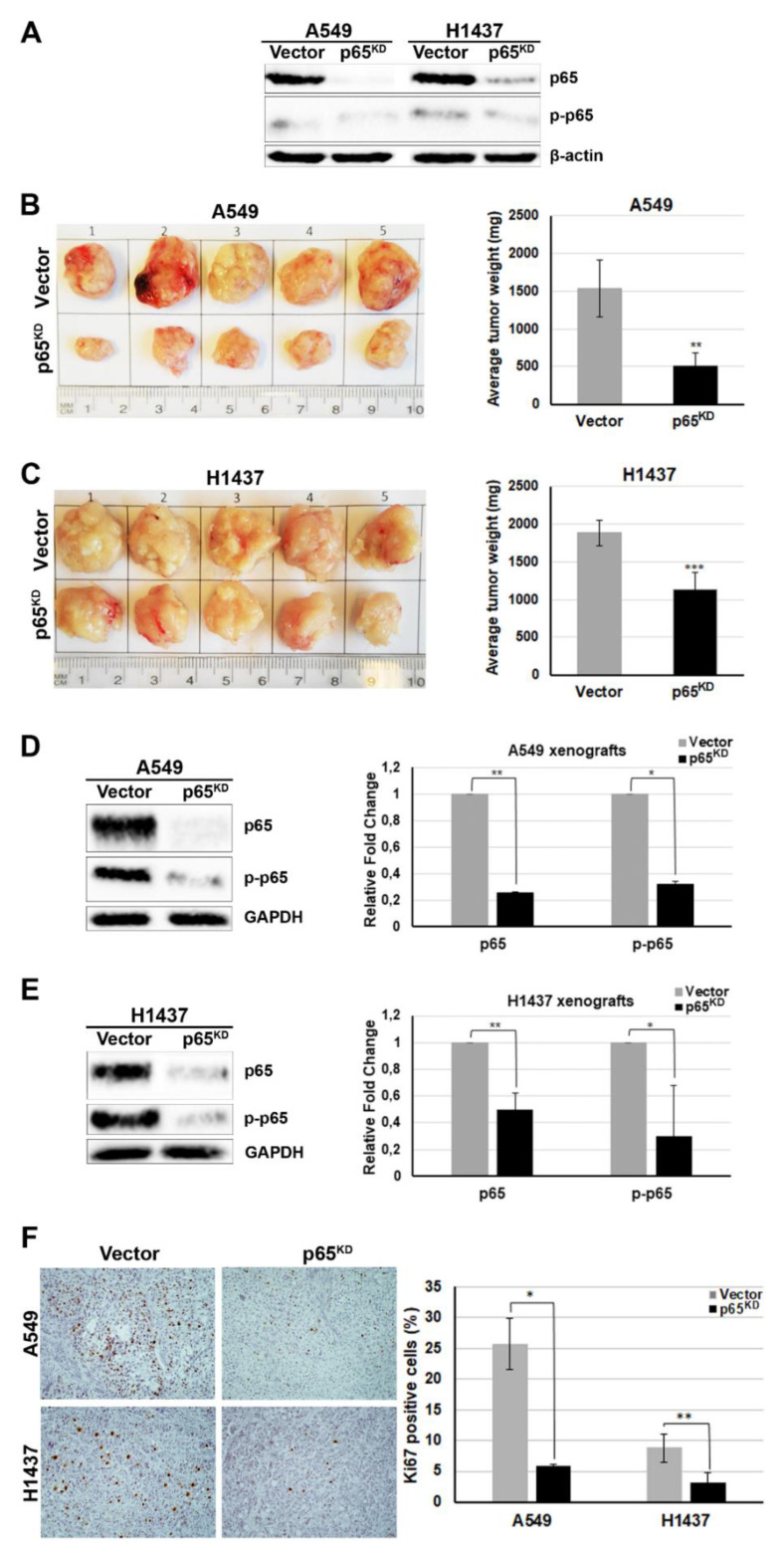
Canonical NF-κB signalling is required for the growth of human NSCLC in murine xenografts. (**A**) Generation of RelA/p65-compromised A549 and H1437 human lung cancer cells. Cells were transfected with either the control vector pSuper-Puro or a pSuper-Puro-sh65 carrying a shRNA against RelA/p65 (p65^KD^) and selected in puromycin to generate stable cell lines. Total proteins extracted were analysed by immunoblotting for the expression of RelA/p65, or β-actin as a reference control. (**B**,**C**) A549 and H1437 cells and their RelA/p65^KD^ derivatives grown in vivo as tumour xenografts. 2 × 10^6^ cells in 200 μL per injection of each of our control and RelA/p65^KD^ A549 and H1237 cells were inoculated subcutaneously into the flanks of NSG mice and maintained for 4 weeks and grown in vivo as tumour xenografts. Comparative photographs of tumour xenografts are shown in the left panels and quantitative analysis of the tumours are shown in the right panels. Significant differences were observed in tumour sizes comparing vector controls to their RelA/p65^KD^ derivatives for each of the two human NSCLC cell lines 4 weeks after their inoculation into immune-compromised mice. H1437 (*n* = 9 mice) *** *p* < 0.005; A549 (*n* = 9 mice) * *p* <0.05 by two-tailed Student’s *t*-tests. (**D**,**E**) Representative immunoblots showing the expression of RelA/p65 in vector control and RelA/p65^KD^ human lung cancer cells A549 (**D**) and H1437 (**E**) grown as tumour xenografts in vivo. Tumours were excised from the animals, and total proteins were isolated and analysed by immunoblotting for the expression of RelA/p65, phospho-p65 (S536) or GAPDH as a reference control (left panels). Quantification of protein expression levels is also provided (right panels) (* *p* < 0.05, ** *p* < 0.01, by two-tailed Student’s *t*-test). (**F**) Paraffin-embedded tissue sections from the excised tumours were analysed by immunohistochemistry for the expression of the proliferation antigen Ki67 (magnification 200X) (*p < 0.05, **p < 0.01, by two-tailed Student’s *t*-test).

A549 and H1437 lung cancer cells were stably transfected with the vectors pS-Puro and pS-Puro-shp65 [28,29] followed by puromycin selection. Immunoblot analysis verified the efficient knock-down of RelA/p65 expression in the stably shp65-transfected human NSCLC cell lines compared to their vector control counterparts (Figure 1A).

Next, we investigated if NF-κB is activated in cultured cells by immunoblotting and luciferase assays by transfecting the A549 and H1437 cells with pGL3, pCMV-Luc and pGL3-5x *κB*-luc reporter plasmids. RelA/p65 was phosphorylated in cultured cells but the expression of the phospho-p65 form was low (Figure 1). Luciferase activity was increased in the cells transiently transfected with the pGL3-5x *κB*-luc reporter compared to pGL3 basic reporter plasmid but it was at much lower levels compared to CMV-driven luciferase expression (Figure S1). Collectively, these data showed that canonical NF-κB was constitutively activated in cultured cells but at low levels.

Next, we analysed the impact of p65^KD^ on cancer cell growth in vitro by constructing growth curves using the IncuCyte live-imaging system. Downregulation of p65 did not impair the proliferation of A549 or H1437 cells grown as monolayers in vitro. Analysis of cell apoptosis showed that p65^KD^ did not affect early or late apoptosis and necrosis (Figure S2).

Next, we investigated the role of RelA/65 in human lung tumour cell growth in vivo and its mechanism of action. To this end, we injected control and RelA/p65^KD^ A549 and H1437 cells into either side of immune-compromised NSG (NOD-SCID-IL2Rgamma) mice and allowed them to grow in vivo as xenografts. RelA/p65^KD^ human NSCLC cell lines presented significantly smaller tumours compared to their wild-type vector control cells. Representative images of dissected tumours grown as xenografts of control A549 and H1437 cells and their RelA/p65^KD^ derivatives are shown, and statistical analyses of tumour weight differences between control and RelA/p65^KD^ tumour xenografts are provided, respectively (Figure 1B,C). This is in agreement with our recent studies showing that IKKβ is required for urethane-induced NSCLC in transgenic mice [26].

To confirm the efficient downregulation of RelA/p65 in vivo, total protein lysates were isolated from the excised tumours and analysed for the expression of p65 and phospho-p65 (S536) by immunoblotting together with statistical analysis (Figure 1D,E). Representative immunoblots are presented showing the expression of RelA/p65 and phospho-p65 (S536) in vector control and RelA/p65^KD^ human lung cancer cells A549 and H1437 grown as tumour xenografts in vivo.

Importantly, NF-κB RelA/p65 was also activated in cells grown as tumour xenografts in vivo, as documented by the expression of the phosphorylated form of RelA/p65, further suggesting that it is required for tumour growth in vivo (Figure 1D).

Immunohistochemical staining of tumour paraffin-embedded sections for the expression of Ki67 proliferation antigen showed that the RelA/p65^KD^ human NSCLC cell lines displayed reduced Ki67 expression compared to vector control counterparts (Figure 1F), indicating that reduced cell proliferation accounts for the decreased tumour growth in vivo in mice.

Taken together, these findings suggest that canonical NF-κB signalling was required for the tumour growth human lung cancer cells in in vivo murine xenografts, hence acting as a tumour promoter of human NSCLC growth.

### 2.2. Up-Regulation of the Metastasis Suppressor CD82/KAI1 and Down-Regulation of the Proto-Oncogene ROS1 in RelA/p65^KD^ Human Tumours Grown in Immune-Compromised Mice

To elucidate the possible molecular mechanisms of action of RelA/p65 in human lung epithelial cancer cells as a NSCLC tumour promoter, we performed a series of unbiased whole gene expression profiling by transcriptome sequencing followed by bioinformatics analysis. Multiple RNA-seq experiments were carried out to compare the transcriptomes of vector control and RelA/p65^KD^ human A549 and H1437 lung cancer cells grown as tumour xenografts to identify differentially expressed transcripts associated with RelA/p65^KD^.

**Figure 2:**
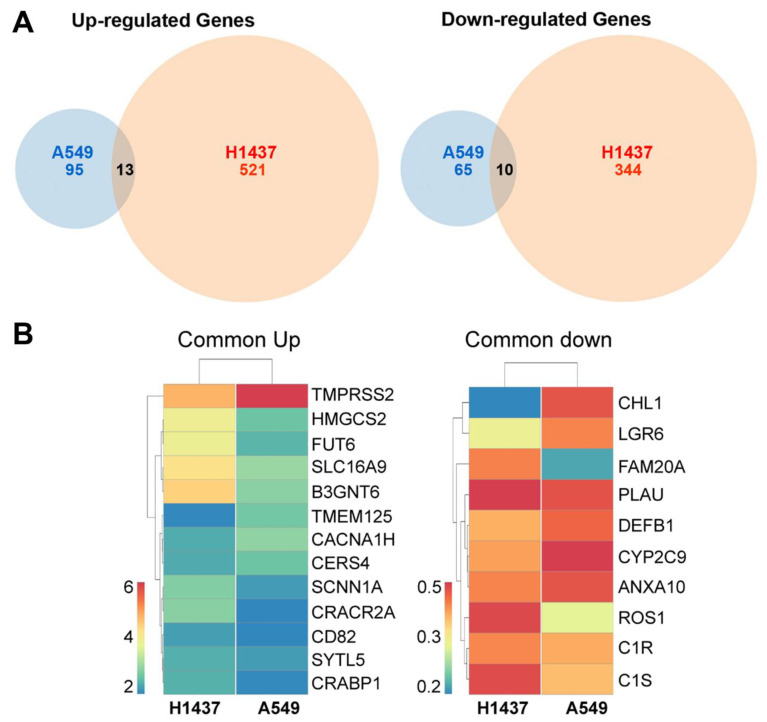
Gene expression profile by multiple RNA-seq experiments (three biological replicates per each cell line) followed by bioinformatics analysis of RelA/p65-compromised human tumours grown as xenografts in immune-compromised mice. (**A**) Following RNA-seq and bioinformatics comparative analysis, Venn diagrams were generated showing 13 genes in common up-regulated, and 10 genes in common down-regulated in both human A549 and H1437 NSCLC tumours; (**B**) heat maps of the common upregulated (left) and downregulated (right) genes in both cell lines that were increased or decreased by at least 2-fold in the tumour xenografts of the RelA/p65^KD^ human A549 and H1437 NSCLC tumours compared to their vector control counterparts.

Venn diagrams and heat maps of Differentially Expressed Genes (DEGs) in the vector control versus RelA/65^KD^ tumour xenografts of human A549 and H1437 NSCLC cells revealed changes in a small number of genes (Figure 2A,B). More specifically, as shown in the Venn diagrams and the heat maps, upon RelA/p65 knock-down, 13 genes in common were upregulated and 10 genes in common were downregulated (*CHL1, LGR6, FAM20A, PLAU, DEFB1, CYP2C9, ANXA10, ROS1, C1R,* and *C1S*) in the A549 and H1437 tumours.

Some of the upregulated genes encode metabolic enzymes such as *HMGSB2* encoding the mitochondrial enzyme 3-hydroxy-3-methylglutaryl-CoA synthase 2, which catalyses the first reaction of ketogenesis, and *CERS4* encoding the enzyme ceramide synthase 4 that catalyses the formation of ceramide from sphinganine and acyl-CoA. Upregulated genes encoding membrane proteins were also identified including *TMPRSS2,* *SLC16A9* (B^0^AT1), *TMEM125, CACNA1H, SCNN1A* and *CRACR2A*, and the first two genes have been involved in SARS-CoV-2 virus entry via ACE2, into airway epithelial cells causing Covid-19 [30,31,32] which is linked to inflammatory responses [33,34,35,36].

Some of the downregulated genes include *CHL1* which encodes the cell adhesion molecule L1CAM2, *CYP2C9* encoding a member of the cytochrome P450 family involved in drug metabolism, and *C1R* and *C1S* encoding components of the complement system C1 complex.

*LGR6* encoding a Leucine Rich Repeat Containing G Protein-Coupled Receptor (GPCR)6, is an epithelial lung stem cell marker involved in the activation of Wnt/β-catenin signalling implicated in the generation and maintenance of cancer stem cells and metastasis [37,38].

Amongst the common upregulated genes was the metastasis suppressor gene *CD82/KAI1* encoding a tetraspanin (TSPAN27) [39,40,41], and amongst the common downregulated genes was the proto-oncogene *ROS1* encoding an orphan type I transmembrane Receptor Tyrosine Kinase (RTK) [42], both of which have been implicated in lung cancer (Figure 2).

### 2.3. Downregulation of p65 Affected the Expression of CD82 and ROS1 in Human Lung Cancer Cells

**Figure 3:**
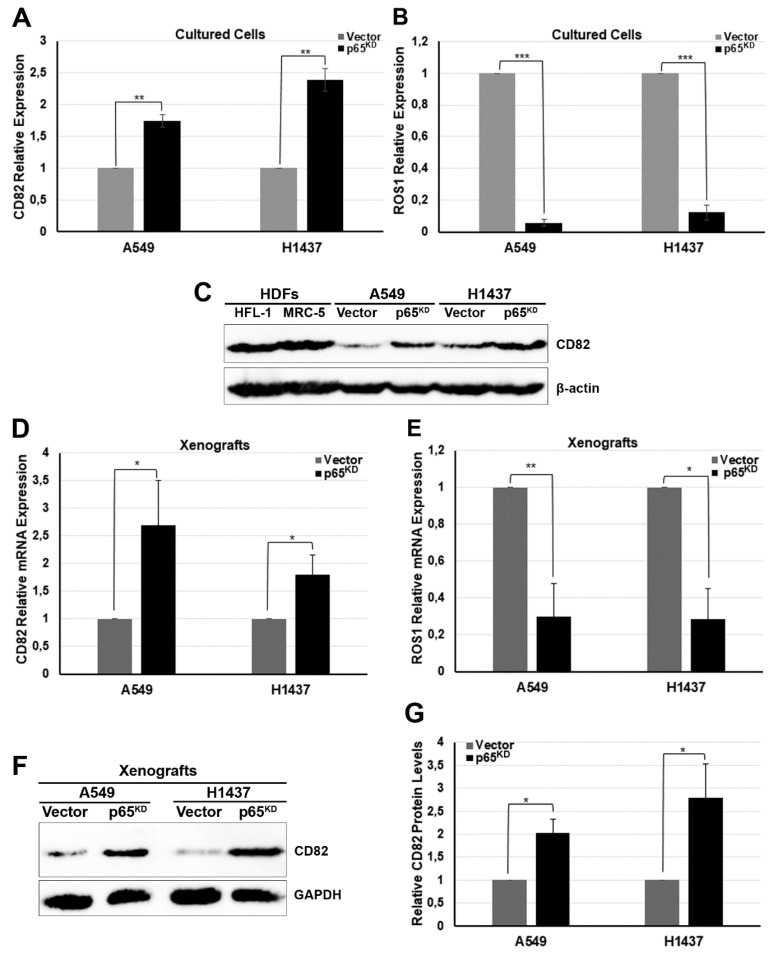
NF-κB p65 regulates the expression of ROS1 and CD82 in vitro and in vivo. (**A**,**B**) Total RNA was isolated from vector control and p65^KD^ A549 and H1437 human lung cancer cells grown in vitro and analysed for the expression of CD82 (**A**) or ROS1 (**B**) mRNA by qPCR (*n* = 3). (**C**) Total protein lysates were isolated from normal lung HDFs, HFL-1 and MRC-5, and from vector control and p65^KD^ A549 and H1437 cancer cells grown in vitro and analysed for the expression of CD82 or β-actin as a reference control by immunoblotting. (**D**,**E**) Total RNA isolated from vector control and p65^KD^ A549 and H1437 cells grown as tumour xenografts in mice was analysed for the expression of (**D**) CD82 and (**E**) ROS1 mRNA by qPCR (*n* = 3). RNA polymerase II was used as the reference control gene. (**F**) Total protein lysates were isolated from vector control and p65^KD^ A549 and H1437 human lung cancer cells grown as tumours in in vivo xenografts in mice and analysed for the expression of CD82 or GAPDH as a reference control by immunoblotting. (**G**) Quantitative analysis of CD82 expression in vector control and p65^KD^ A549 and H1437 cancer cells grown in vivo (*n* = 3). All statistical analysis is a result of three biological replicates using a two-tailed Student’s *t*-test (* *p* < 0.05, ** *p* < 0.01, *** *p* < 0.001, bars represent mean ± SD). The panels in Figure 3F resulted from the joining of strips of different blots. For all quantification analysis raw, unprocessed images were used.

To experimentally validate the changes in expression of CD82 and ROS1, total RNA isolated from vector control and p65^KD^ A549 and H1437 cancer cells grown in culture were analysed by real-time qPCR (Figure 3). Loss of RelA/p65 in human NSCLC cells resulted in the upregulation of CD82/KAI1 (Figure 3A) and the downregulation of ROS1 proto-oncogene (Figure 3B) mRNA levels. The protein expression levels of CD82 in cultured cells was also investigated by immunoblotting and compared to HFL-1 and MRC-5 HDFs. RelA/p65^KD^ resulted in the upregulation of CD82 protein expression to levels similar to those detected in normal HDFs (Figure 3C).

Next, we investigated the expression of CD82 (Figure 3D) and ROS1 (Figure 3E) mRNA by real-time qPCR in vector control and RelA/p65^KD^ A549 and H1437 cancer cells grown as tumour xenografts in mice. Loss of RelA/p65 in human NSCLC cells grown as tumours in vivo lead to the upregulation of CD82/KAI1 and to the downregulation of ROS1 proto-oncogene mRNA levels. The protein expression levels of CD82 in the tumours were also investigated by immunoblotting. Loss of p65 resulted in the upregulation of CD82 protein levels in both human NSCLC lines (Figure 3F,G). Collectively, these data confirmed that both CD82/KAI1 and ROS1 are RelA/p65 targets.

### 2.4. Decreased Expression of CD82/KAI1 in Human Lung Cancer Tissues

Initially, we analysed the expression of CD82/KAI1 in whole sections of 16 patients with LUAD and 13 patients with LUSC. It was found that expression of CD82/KAI1 was decreased with the progression of human lung cancer (Figure S3A).

**Figure 4:**
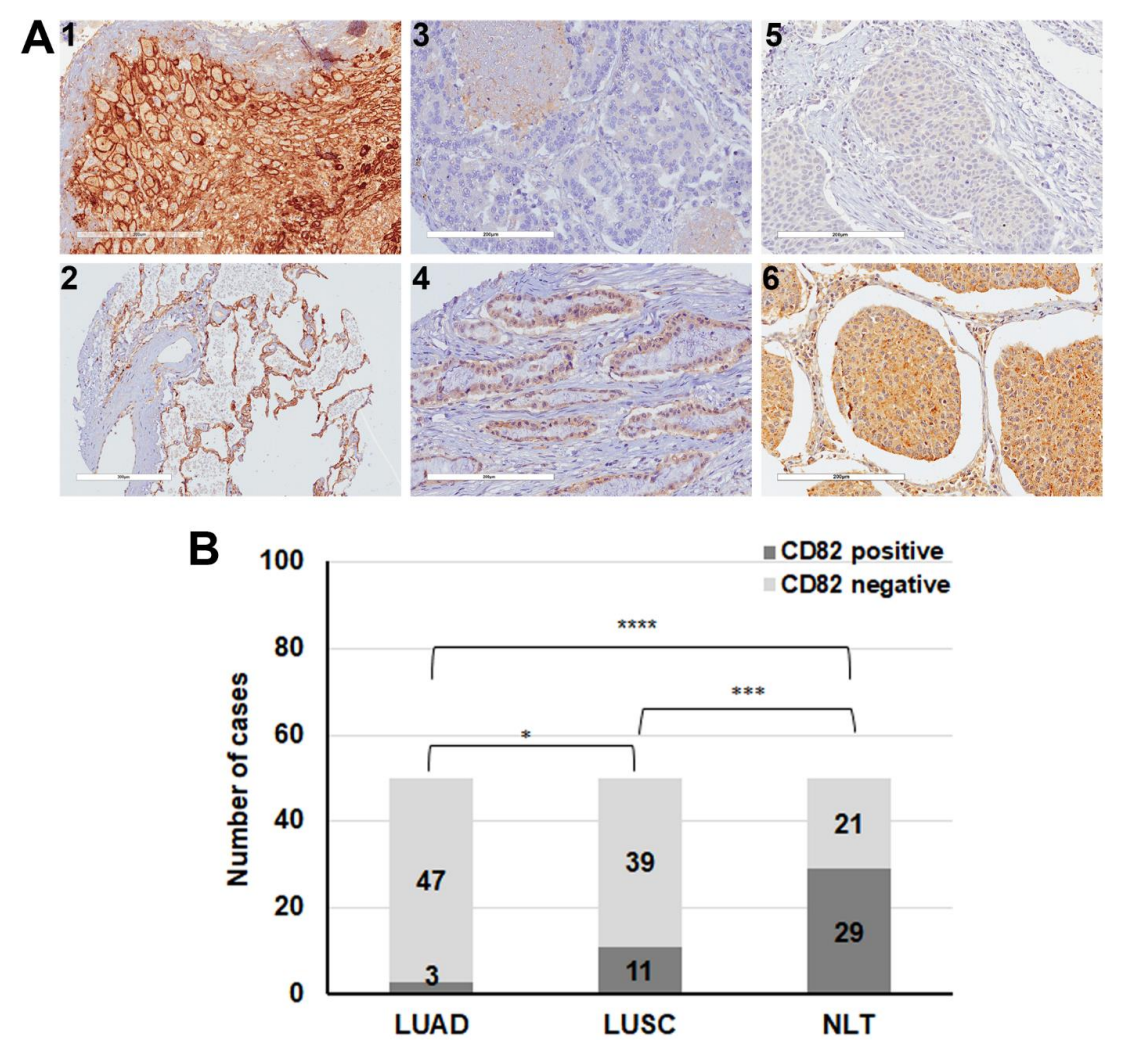
Expression CD82/KAI1 expression in normal and tumour human lung tissue samples. (**A**) Analysis of CD82 expression in normal human lung tissue (NLT) (*n* = 50) and patient-derived human LUAD (*n* = 50) and LUSC (*n* = 50) tissue microarrays (TMAs) by immunohistochemistry. **1** Placenta tissue sample used as a positive control for CD82 expression. **2** Normal human lung tissue showing expression of CD82 localised in the plasma membrane of lung epithelial cells, **3** LUAD negatively stained for CD82. **4** LUAD exhibiting cytoplasmic staining of CD82 in the tumour cells. **5**, **6**, LUSC negatively and positively stained for CD82, respectively. (Scale 1, 3, 4, 5, 6, 200 μm, and 2, 300 μm). (**B**) Statistical analysis of CD82 expression in TMAs of normal human lung tissue and in lung tissues of patients with LUAD and LUSC by Fisher’s exact test (* *p* < 0.05, *** *p* < 0.001, **** *p* < 0.0001).

CD82 expression was analysed by immunohistochemistry in tissue microarrays (TMAs) that consisted of normal lung tissue (NLT), and samples of patients with LUAD and LUSC (Figure 4A). CD82 expression was significantly decreased in tumour versus normal lung tissue (*p* < 0.001), while there was also a significant difference between tumour types as only 6% of the LUAD samples were found positive for CD82 expression compared to 22% of LUSC patient samples (*p* < 0.05) (Figure 4B).

In addition to our results, bioinformatics analysis showed that CD82 is significantly downregulated in LUAD (*p* < 0.05) and in LUSC (albeit non-significantly) compared to the normal lung tissue (GTEX) (Figure S3B), in both males and females suggesting no correlation between CD82 expression and the sex of the patients (Figure S3C). There was no correlation between CD82 expression and the stage of the NSCLC patients (Figure S3D), and also based on the staining of whole sections of early and advanced stage NSCLC (data not shown), suggesting that once malignancy is established, CD82 expression is downregulated with a small and insignificant reduction with disease progression.

Recent studies showed that CD8^+^ tissue infiltrating lymphocytes (TILs) is the best candidate marker for immune cells in NSCLC, with a prognostic efficacy in both LUAD and LUSC [43]. Hence, we investigated the presence of CD8^+^ TILs in a set of 29 NSCLC patient’s whole sections. CD8^+^ TILs were increased in the intra- and peri-tumoural compartment of CD82 negative tumours. However, this difference did not reach statistical significance (Figure S4 and Table S1).

To gain a better insight of immune cells in NSCLC, we expanded our observations on single cell analysis of human immune cells in lung cancer using the Single Cell Portal [44]. Using T-distributed Stochastic Neighbour Embedding (tSNE)—a machine learning algorithm for visualisation—distinct cell sub-populations appear to express CD82 (Figure S5A). Specifically, sporadic expression was noted in neutrophils, T-cells, B-cells and mast cells (Figure S5B).

### 2.5. Downregulation of p65 Reduced Cell Migration and Enhanced the Epithelial Cell Phenotype

**Figure 5:**
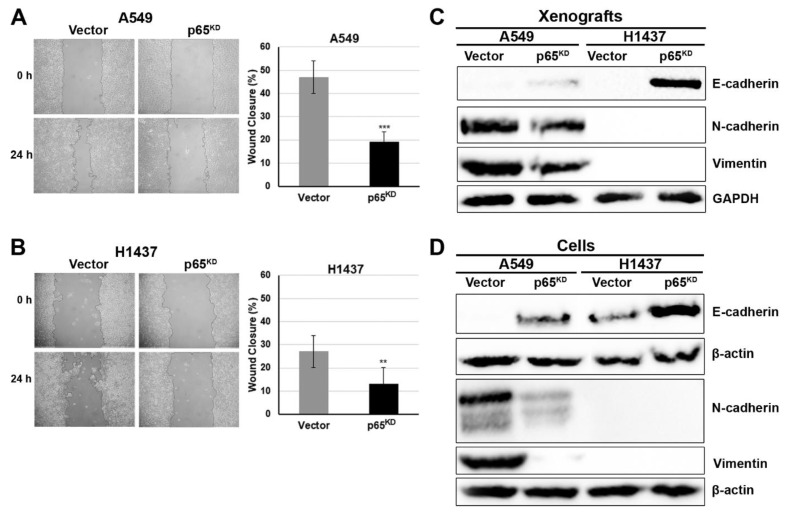
Loss of p65 reduces cell migration and enhances the expression of an epithelial cell phenotype. (**A**,**B**) In confluent monolayer cultures of vector control and p65^KD^ A549 and H1437 human lung cancer cells a scratch was generated, and the cells were allowed to heal the wound by establishing new cell-cell contacts. (**C**,**D**) Loss of RelA/p65 enhances the epithelial phenotype. Total protein lysates were extracted from vector control and p65^KD^ A549 and H1437 cancer cells either grown as tumours in in vivo xenografts in NSG mice (**C**) or cultured as monolayers in vitro (**D**), and analysed by immunoblotting for the expression of E-cadherin, N-cadherin and vimentin or GAPDH and β-actin as a reference control. The panels in Figure 5D resulted from the joining of strips of different blots. For all quantification analysis raw, unprocessed images were used. ** *p* < 0.01, *** *p* < 0.001.

Because loss of RelA/p65 resulted in a significant decrease in tumour growth and the induction of the metastasis suppressor CD82, we next investigated the impact of p65 on cell migration in vitro using the wound healing assay. In confluent monolayers of control and p65^KD^ A549 and H1437 cancer cells a scratch was generated and the cells were allowed to heal the wound by establishing new cell-cell contacts. Loss of p65 in both A549 (Figure 5A) and H1437 (Figure 5B) cancer cells profoundly reduced cell migration compared to vector control cells.

The reduction in cell migration prompted us to investigate the effects of p65 on the expression of the epithelial cell phenotype. Loss of an epithelial phenotype in cancer is observed during an epithelial-to-mesenchymal cell transition (EMT), a hallmark of cancer detected in metastasising cancer cells. EMT is characterised by the expression of certain biomarker proteins such as loss of the epithelial cell marker E-cadherin and increased expression of mesenchymal cell markers such as N-cadherin and vimentin [45,46,47,48,49,50].

Canonical NF-κB was shown to regulate EMT at several levels [50,51,52,53,54,55]. Hence, we investigated if loss of p65 affected the expression of both epithelial and mesenchymal cell markers. Loss of RelA/p65 resulted in the induction of E-cadherin expression in both A549 and H1437 cells and in the suppression of N-cadherin and vimentin in A549 cells in both cells grown as tumour xenografts (Figure 5C), or cultured as monolayers (Figure 5D). H1437 cells expressed undetectable levels of N-cadherin and vimentin, under our growth conditions, in agreement with previous studies [56,57]. The expression and localisation of E-cadherin were also investigated by immunofluorescence in vector and RelA/p65^KD^ H1437 cells. Loss of RelA/p65 resulted in a marked increase in cell surface expression of E-cadherin compared to their control counterparts (Figure S6).

### 2.6. Downregulation of p65-Reduced Cell Migration and EMT Was Due to Induction of CD82

**Figure 6:**
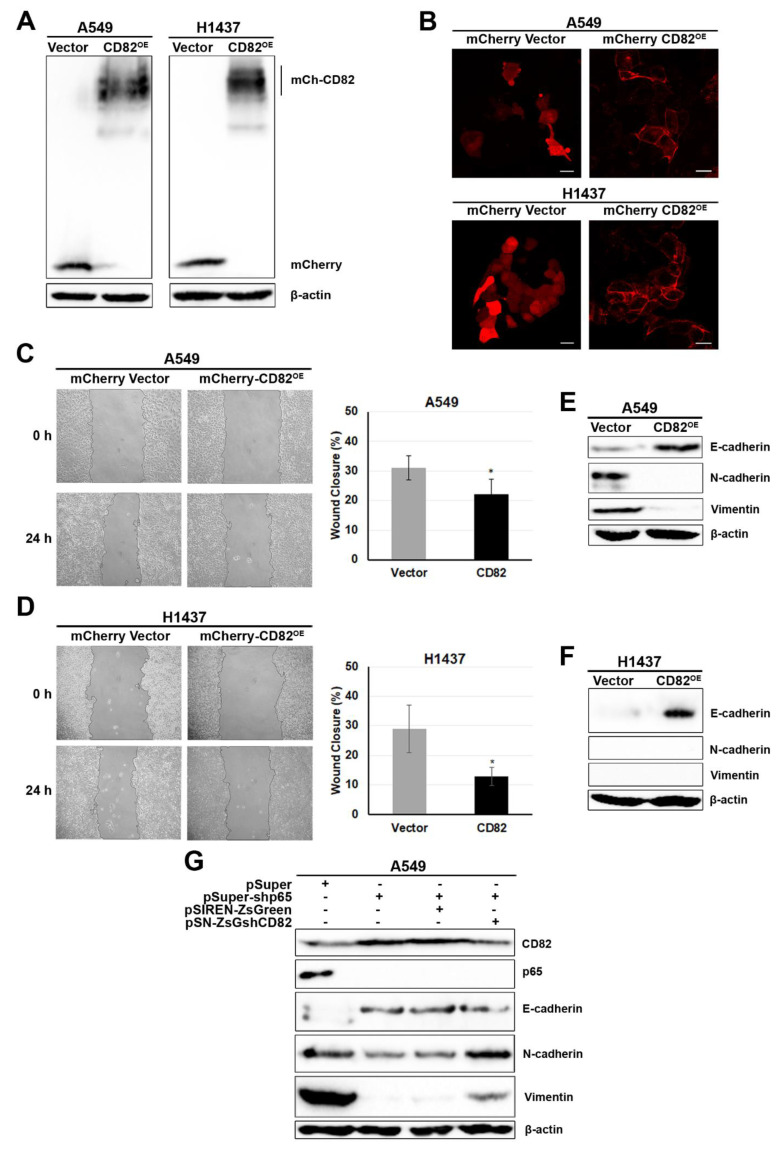
Effects of p65 and CD82 on cell migration and the expression of EMT markers in human lung cancer cells. (**A**) Generation of CD82-overexpressing human lung cancer cells. Control mCherry and mCherry-CD82^OE^ (mCh-CD82^OE^) vectors were transfected in A549 and H1437 cells following selection in G418 to generate stable cell populations overexpressing the mCherry-CD82 fusion protein. (**B**) Cytoplasmic localisation of mCherry protein in vector control mCherry cells compared to membrane localisation in mCh-CD82^OE^ A549 and H1437 cells detected by fluorescence microscopy. (**C**,**D**) In confluent monolayers of mCherry vector control and mCherry-CD82^OE^ in A549 (**C**) and H1437 (**D**) cells, a scratch was generated and the cells were allowed to heal the wound by establishing new cell-cell contacts. The percent of wound closure was estimated and presented as bar graphs on the right-hand side of (**C**,**D**) for A549 and H1437 cells, respectively (* *p* < 0.05 by Student’s *t* test, bars represent ±SD). (**E**,**F**) Overexpression of CD82 (CD82^OE^) enhances the epithelial phenotype. Total protein lysates were extracted from vector control and CD82^OE^ A549 (**E**) and H1437 (**F**) cancer cells and analysed by immunoblotting for the expression of E-cadherin, N-cadherin and vimentin or β-actin as a reference control. (**G**) p65^KD^ A549 cancer cells were infected with either the control retroviral vector or a vector carrying a ShCD82. The retroviral vectors used were the control pSIREN-ZsGreen or pSIREN-ZsGreen-shCD82 (pSN-ZsG-sh82) expressing a CD82-specific shRNA in addition to fluorescence protein ZsGreen, as indicated at the top. Total protein lysates were extracted and analysed by immunoblotting for the expression of CD82, p65, and for the epithelial and mesenchymal cell markers E-cadherin, N-cadherin and vimentin, or β-actin as a reference loading control.

CD82 has been shown to act as a metastasis suppressor through various mechanisms [39,41,58], and since RelA/p65^KD^ cells exhibit reduced cell migration and an enhanced epithelial phenotype, we investigated the impact of CD82 on cell migration and EMT. We generated CD82-overexpressing A549 and H1437 cancer cells by transfection with either a control mCherry expression vector or the same vector carrying CD82 fused to mCherry (mCherry-CD82^OE^) [59,60], followed by selection in G418 and FACS sorting. CD82 expression was verified by immunoblotting using a mCherry-specific antibody. A 45 kDa mCherry protein was detected in the mCherry vector control cells, whereas a 70 kDa protein was detected in the mCherry-CD82^OE^-transfected cells due to mCherry-CD82 protein fusion. Higher, heterogeneous molecular weight CD82 proteins detected were most likely due to N-linked glycosylations involved in CD82 functions [61,62] (Figure 6A). Importantly, expression and localisation of the fused mCherry-CD82 protein was detected in the plasma membrane of the mCherry-CD82^OE^ A549 and H1437 cells as visualised by fluorescence microscopy (Figure 6B). CD82 did not significantly affect the proliferation of mCherry-CD82^OE^ A549 and H1437 cells compared to their vector control counterparts (Figure S7).

Next, we performed a scratch assay to measure the impact of CD82 on cell migration in vitro. Overexpression of CD82 in cancer cells markedly reduced their migration ability/capacity compared to mCherry-vector control A549 (Figure 6C) and H1437 (Figure 6D) cells.

We also analysed the specific expression of epithelial and mesenchymal cell markers, by immunoblotting (Figure 6E,F). CD82^OE^ resulted in the induction of E-cadherin expression in both A549 and H1437 cells and in the suppression of N-cadherin and vimentin in A549 cells (Figure 6E,F). H1437 cells expressed undetectable levels of N-cadherin and vimentin, (Figure 6F).

To further confirm our results that the effects of p65^KD^ on the expression of the epithelial cell phenotype were mediated through CD82, we knocked-down the expression of CD82 in RelA/p65^KD^ A549 cancer cells (Figure 6G) using the control retroviral vector pSIREN-ZsGreen or pSIREN-ZsGreen-shCD82 [63] (Figure S8). The simultaneous downregulation of p65 and CD82 was verified by immunoblotting in the double-transfected cells (Figure 6G). Total protein lysates from A549 vector control, p65^KD^ and p65^KD^/CD82^KD^ cells were also analysed for the expression of E-cadherin, N-cadherin and vimentin. Downregulation of CD82 in p65^KD^ cells was able to restore N-cadherin protein expression levels and to a lesser extent the expression levels of E-cadherin and vimentin indicating that p65 acts in favour of EMT progression in part through downregulation of CD82.

### 2.7. RelA/p65^KD^ Impaired Integrin-Mediated Signalling Involving ERK, Akt and Rac1

**Figure 7:**
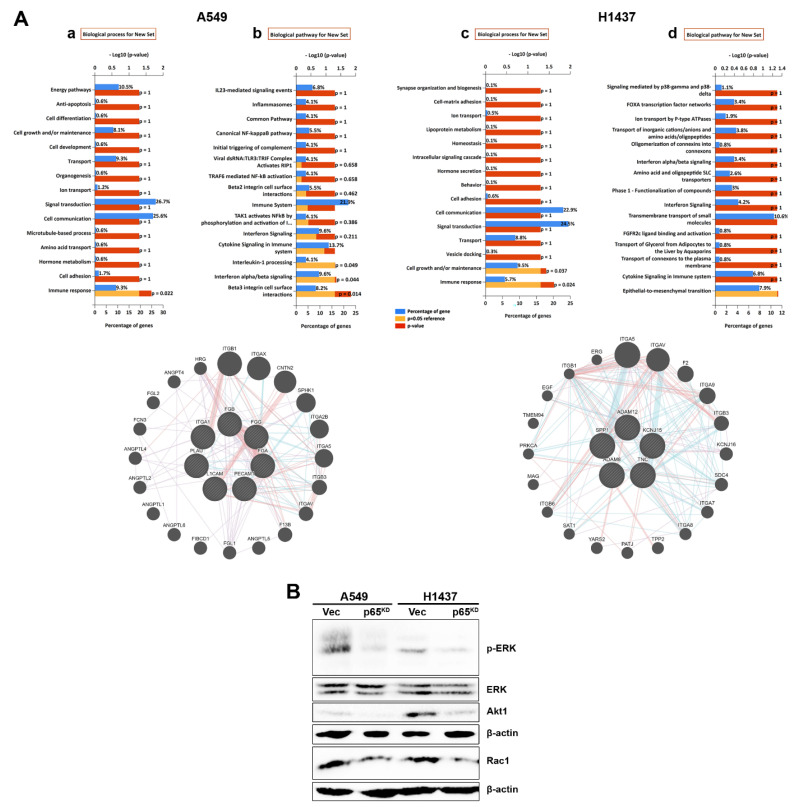
Effects of p65 on integrin-mediated signalling in human lung cancer cells. (**A**) Bioinformatics analysis of NF-κB RelA/p65-regulated genes involved in a particular pathway. Panels (**a**,**b**) show the biological processes and the percentage of genes of the entire cohort involved in a particular process. Panels (**c**,**d**) show the biological pathways affected. The analysis of all genes in the human A549 and H1437 NSCLC cell lines revealed that genes involved in immune response, integrin pathways and epithelial-to-mesenchymal cell transition were affected. The red/yellow bars represent the *p*-value. The more yellow, the more significant the enrichment. The role of the enrichment is to identify processes that can be potentially altered based on all differentially expressed genes. All genes involved in integrin pathways for A549 and H1437 and for “α9β1 integrin signalling events” were used to model gene-gene interactions revealing an interesting crosstalk. (**B**) Expression of protein molecules involved in integrin-mediated signalling, such as ERK, phospho-ERK, Akt1 and Rac1 in vector control and p65^KD^ A549 and H1437 human lung cancer cells.

The downregulation of E-cadherin and the reduced cell migration in the absence of RelA/p65 prompted us to investigate the engagement of signalling pathways by bioinformatics using the NCI-Nature pathway analysis. A link between NF-κB RelA/p65, CD82 and integrin signalling pathways was revealed. The analysis identified similarly affected pathways in both cell lines, A549 and H1437. For example, in the biological processes, 9.3% and 5.7% of genes of the entire cohort, for A549 and H1437, respectively, were involved with the immune responses (Figure 7Aa,c). In the biological pathways, genes involved in integrin interactions and epithelial-to-mesenchymal transition were affected (Figure 7Ab,d), and the involvement of integrin-mediated signalling was also verified (Table S2).

Based on these data, next we investigated the expression of several protein molecules involved in integrin-mediated signalling [64] in vector control and p65^KD^ A549 and H1437 human lung cells. Loss of Rel/p65 in both A549 and H1437 cell lines resulted in the downregulation of phospho-ERK, Akt1 and Rac1 downstream signalling molecules (Figure 7B), in agreement with the bioinformatics analysis (Figure 7A and Table S2).

## 3. Discussion

Canonical NF-κB signalling components such as the IKKβ kinase and RelA/p65 have been shown to act as tumour promoters in several models of NSCLC. These include the chemical carcinogen urethane-induced NSCLC model in mice [25,26] and the oncogene-induced NSCLC, such as mutant oncogenic *KRAS* [11,12,13,14] and *EGFR* [15,16,17], both in mouse NSCLC transgenic models and in human NSCLC cell lines transplanted in immune-compromised mice.

Human NSCLC is initiated by tumour-initiating cells (TICs) bearing specific mutations giving rise to different histological subtypes such as LUAD and LUSC [3,4,6]. Using genetic approaches to inducibly express *KRAS*^G12D^ in CC10^+^ and Sftpc^+^ AT-II epithelial cells of the adult mouse lung, it was found that AT-II and Clara cells in the terminal bronchioles, and bronchoalveolar stem cells were identified as cells of origin for K-Ras^G12D^-induced lung hyperplasia and carcinomas, but only AT-II cells were identified as the predominant cell of origin of LUAD induced by K-Ras^G12D^ activation [8,9,65,66]. Importantly, lung cancer development and progression is also facilitated by the microenvironment surrounding the TICs. LUAD promotion is fuelled by inflammation leading in enhanced pneumonocyte proliferation [21], and it is reduced by IKKβ ablation in myeloid cells [14].

A549 and H1437 NSCLC cell lines were used to generate RelA/p65-compromised derivatives and investigate its impact on tumour growth and its mechanism(s) of action. While downregulation of RelA/p65 did not affect the proliferation of the human lung cancer cells grown as monolayers in vitro, it was required for their tumour growth in vivo grown as xenografts in immune-compromised mice (Figure 1), suggesting that RelA/p65 functions in lung epithelial cells as an NSCLC tumour promoter. In general, the rate of cancer cell proliferation in vivo is much slower than that in vitro, and tumour growth does not depend solely on cell proliferation but on several other factors including cell adaptation, stroma cell recruitment, the establishment of cell matrix interactions, the microenvironment in vivo, and to overcome species differences [67,68]. RelA/p65, being a regulator of a plethora of genes, may therefore regulate some of these factors and contribute to tumour growth in vivo. Our data is in agreement with the findings in urethane- and the oncogene-induced mouse NSCLC models wherein expression of an IκBαSR super-repressor [11,25] or deletion of RelA/p65 [12] lead to impairment of tumour growth. Similarly, conditional deletion of IKKβ reduced cancer cell proliferation and tumour burden of adenocarcinomas in both urethane- [26] and *KRAS*^G12D^- [13] induced mouse NSCLC, suggesting that IKKβ was also required for LUAD development in vivo.

Transcriptome analysis of vector control and Rel/p65^KD^ A549 and H1437 cell tumours identified the proto-oncogene *ROS1* and the *LGR6* receptor gene amongst the downregulated genes, and the metastasis suppressor *CD82* as one of the upregulated genes. We verified that Rel/p65^KD^ resulted in the downregulation of the *ROS1* and in the upregulation of CD82 mRNA levels (Figure 2).

*ROS1*, which encodes a receptor tyrosine kinase with no known ligands, is activated in ~1–2% of NSCLC by gene rearrangements resulting in novel chimeric fusion proteins, SLC34A2-ROS and CD74-ROS [42]. However, as the human ROS1 gene promoter lacks *κB* elements and not all human lung cancer cell lines express ROS1 protein, including A549 and H1299 which are fusion-negative cell lines [69,70], we further investigated the role of CD82 [40] in epithelial lung cancer cell growth.

A few studies have identified canonical NF-κB targets accounting for its tumour promoting function in NSCLC. For example, an NF-κB-regulated genetic signature identified in response to mutant *EGFR* oncogene inhibition in human NSCLC cells consisted of 36 genes, including the cell survival genes *TNFAIP3*, *BIRC3* and *IL-6* [16]. In a K-Ras^G12D^-induced LUAD mouse model, Timp1 was identified as a IKKβ-mediated canonical NF-κB-regulated tumour growth regulator. Further, it was shown that activation of ERK signalling and cell proliferation required Timp1 and its receptor, the tetraspanin CD63. Knocking-down IKKβ or Timp1 reduced tumour growth in xenografts [13]. Thus, Timp1-CD63 regulates epithelial cell proliferation and apoptosis. CD82 was identified as a component of the cell surface TIMP1-interacting protein complex by directly binding to Timp1 amino-terminal region through its large extracellular loop domain and, it was shown to co-localise with Timp1 in cancer cell lines and tumour tissues. CD82 was shown to facilitate membrane-bound Timp1 endocytosis, significantly contributing to the anti-motility effects of TIMP-1 [13]. Hence, it is important to emphasise that our RelA/p65 gene signature identified CD82 involved in NSCLC growth. Although the human *CD82/KAI1* gene promoter contains NF-κB binding sites, and NF-κB was shown to regulate CD82 expression with a dependency on the extracellular signals leading to the recruitment of co-activators or co-repressors [40,71,72,73], the mechanisms regulating CD82 transcription are still controversial [62]. Previous studies also showed that serine phosphorylated RelA/p65 can repress specific promoters, such as the tumour metastasis suppressor gene BRMS1, by recruiting the DNMT-1 methylase [74]. Anti-inflammatory responses leading to the downregulation of cytokine signalling also induce the expression of CD82 in a human lung carcinoma cell line [75].

The CD82/KAI1 metastasis suppressor gene encodes tetraspanin TSPAN27, a transmembrane glycoprotein that is a member of the transmembrane 4 superfamily. It regulates T-cell signalling through the T-cell receptor (TCR). It associates with CD4 or CD8 and delivers costimulatory signals for the TCR/CD3 pathway. Expression of CD82 is downregulated during tumour progression of human cancers [39,40,41,62]. CD82 expression was markedly decreased in LUAD and LUSC versus normal lung tissue, but more profoundly in the former (Figure 4). The transition from normal or benign stage to a malignant stage was accompanied by a profound reduction in the expression of CD82; but loss of CD82 expression appeared to be independent of disease progression, which was also confirmed by bioinformatics analysis of the data sets TCGA and GTEX (Figure S2), in agreement with previous studies [39,40,41,62,76]. CD82 did not appear to be a valid biomarker of overall survival, although low CD82 expression in LUAD appeared to be associated with poorer survival (not shown). Because CD82 acts as a metastasis suppressor and interacts with CD8, although we showed that CD8^+^ TILs were increased in CD82 negative tumours, this difference was not statistically significant, suggesting that the suppressing activity of CD82 may not be related to the recruitment of CD8^+^ TILs (Figure S4 and Table S1).

The progression of cancer cells to invasive, metastatic cells involves an “invasion-metastasis” cascade and the activation of the EMT programme. This is associated with the loss of an epithelial cell phenotype and the acquisition of a mesenchymal cell-like cell phenotype, which is crucial for cancer cell migration, the invasion of adjacent tissues and metastasis, that is dissemination via the circulation into distant tissues/organs and colonisation [45,46,47,48]. The metastatic process is fuelled by inflammation, a hallmark of cancer, leading to the activation of canonical NF-κB, a major regulator of pleiotropic EMT-inducing transcription factors (EMT-TFs), such as Snail/SNAI1, Slug/SNAI2 and Twist [50]. Activation of the EMT-TFs leads to the loss of the epithelial cell marker E-cadherin, and the induction of the expression of mesenchymal cell proteins, such as N-cadherin, vimentin, fibronectin, and matrix metalloproteinases (MMPs) [46,50]. Integrins are transmembrane proteins which interact with both extracellular matrix and intracellular cytoskeletal molecules, and activate several signalling pathways involved in cell adhesion, motility and growth, and promote tumour metastasis [64]. EMT and cell migration can also be promoted by integrin signalling which activates Rac1 and canonical NF-κB signalling [64].

We showed that loss of p65 reduced cell migration and enhanced the expression of the epithelial cell marker E-cadherin, and decreased the expression of the mesenchymal cell markers, N-cadherin and vimentin (Figure 5 and Figure S5), an effect mediated, in part, by CD82 in support of its metastasis suppressor function, since forced expressions of CD82 suppressed cell migration and EMT (Figure 6), in agreement with previous studies [77,78,79].

Bioinformatics analysis linked p65-CD82 to integrin signalling (Figure 7 and Table S2). Previous studies showed that CD82 interacts with many membrane proteins and acts to suppress metastasis via several different mechanisms [39,40,41,62]. CD82 was shown to physically interact with other proteins and also to indirectly affects critical signalling pathways through phosphorylation-mediated activation of proteins. It was shown that CD82 associates with the EGFR and integrins, including α3, α4, α5, α6 and β1 accelerating their co-internalisation and resulting in reduced cell migration [39,40,58,59,76,80,81]. CD82 expression was also associated with decreased β-catenin degradation, leading to its accumulation at the plasma membrane and the stabilisation of cell surface E-cadherin-β-catenin complexes which promote cell-cell adhesion and restrain metastasis [58,82]. This is interesting as a β-catenin/Reptin complex was found to represses CD82 transcription and also NF-κB activation [73], suggesting the existence of a feed-forward mechanism whereby an increase in β-catenin inhibits CD82 expression to enhance Wnt signalling and cancer metastasis [40,83].

We investigated several downstream effector molecules of integrin-mediated signalling and showed that loss of RelA/p65 results in the downregulation of phospho-ERK, Akt1 and Rac1 involved in cell proliferation, survival and motility [64,80]. Thus, p65-CD82 functions by suppressing integrin-mediated EMT, cell migration and tumour growth. The metastasis suppressor function of CD82 was first documented in a rat prostate cancer model [84], and also in a murine orthotopic lung cancer model [85], and in several other cancer cell models including hepatocarcinoma, melanoma, sarcoma, pancreatic and breast cancer affecting in vivo invasion and metastasis [40]. The tumour suppressive actions of CD82 are due to several different mechanisms, including interference with integrin-mediated signalling through direct interactions with integrin subunits [39], but also by indirectly affecting other signalling pathways [39,86].

The changes in the EMT programme and cell migration associated with the loss of RelA/p65, in conjunction with the changes expression of several cell surface genes may be related to the plasticity cell states recently described in human LUAD tumours [8,9]. This is further supported by our finding that LGR6 was downregulated upon loss of RelA/p65, most likely resulting in the suppression of Wnt/β-catenin signalling implicated in the induction of cancer stem cells and metastasis, suggesting that this may be another mechanism by which canonical NF-κB modulates Wnt signalling, cancer stem cell expansion and metastasis of lung cancer cells [37,38]. Importantly, CD82 suppresses β-catenin-mediated Wnt signalling activation and significantly reduces β-catenin levels through the exosomal clearance of β-catenin [40,87].

Collectively, these data support our RelA/p65 gene signature in human lung cancer cells, and provides a mechanism by which canonical NF-κB signalling contributes to NSCLC development and progression.

## 4. Materials and Methods

### 4.1. Cell Culture

Normal human lung fibroblasts (HDFs), HFL-1 and MRC-5 [88,89,90] and the human NSCLC cells A549 (K-Ras^G12S^, p53^wt^) were cultured in low glucose Dulbecco’s modified Eagle medium (DMEM), and H1437 (K-Ras^wt^, p53^R247^) in RPMI-1640 [26]. The retrovirus packaging cell line, Phoenix, was cultured in high glucose DMEM (Sigma-Aldrich Chemical Co., St. Louis, MO, USA). All culture media were supplemented with 10% foetal bovine serum (Gibco-Thermo Fisher Scientific, Waltham, MA, USA), 2 mM L-glutamine, 100 units/mL penicillin and 100 μg/mL streptomycin (Biowest, Nuaillé, France). Cells were incubated at 37 °C, 5% CO_2_.

### 4.2. DNA Transfections

A549 and H1437 cells were transfected with the plasmid vectors pSuper-Puro (pS-Puro) and pSuper-Puro-shp65 (pS-Puro-shp65) [28,29] using polyethilenimine (PEI linear, MW25000; Polysciences Europe GmbH). For the overexpression of CD82, A549 and H1437 cells were transfected with the plasmid vectors mCherry-C1 and mCherry-CD82^OE^ [59,60]. Briefly, 4 μg of plasmid DNA was mixed with 30 μg PEI in 100 μL serum- and antibiotic-free DMEM and incubated for 10 min at room temperature, and then added to ~70% confluent cells for 24 h. The transfection medium was removed, the cells were washed twice with serum-free DMEM and incubated for another 24 h before applying selection in 3 μg/mL puromycin for 10 days to generate stable RelA/p65 knockdown (RelA/p65^KD^) cell lines [26,88]. For the stable generation of CD82^OE^ cell lines, the transfected cells were selected in 500 μg/mL G418 followed by sorting based on red fluorescence.

### 4.3. Retroviral Vectors and Infections

The retroviral vectors used were the control pSIREN-RetroQ-ZsGreen (pSIREN-ZsGreen) and pSIREN-RetroQ-ZsGreen-shCD82 (pSIREN-ZsGreen-shCD82) expressing a CD82-specific shRNA [63]. Cells were infected with high-titre control retroviruses or carrying shCD82, generated following transfection of amphotropic phoenix cells [89,90]. Polyclonal populations of stable retroviral transduced cells were obtained by GFP selection.

### 4.4. Assessment of Cell Proliferation and Apoptosis of Human Lung Cells

Equal number of control vector and p65^KD^ cells were plated in 24-well plates and monitored by the IncuCyte-ZOOM^®^ Live-Cell Analysis System (Sartorius, Michigan, MI, USA) for 92 h to construct growth curves using the IncuCyte^®^-ZOOM software.

Cell apoptosis was assessed by Annexin V-FITC/PI staining kit (Biolegend #640914, San Diego, CA, USA) according to the manufacturer’s instructions, and analysed with a fluorescence activated cell sorter (BD FACSAria III, BD Biosciences, San Jose, CA, USA). The percentages of early (Annexin V^+^) and late (Annexin V^+^/PI^+^) apoptotic cells in each group were determined and represented in bar graphs.

### 4.5. Luciferase Assays

For NF-κB luciferase reporter assays, cells were seeded at 60% confluent in 12-well plates and transfected with Polyethylenimine (PEI) (Polysciences Europe GmbH). The NF-κB luciferase reporter assays were performed using the Dual-Luciferase Reporter Assay System (Promega E1910) which measures sequentially the activities of firefly (*Photinus pyralis*) and *Renilla* (*Renilla reniformis* or sea pansy) luciferases from a single sample. Briefly, cells were transiently transfected with 0.3 μg/well of a luciferase reporter plasmid pGL3, pCMV-Luc (Addgene #45968, Watertown; MA, USA) or pGL3-5x *κB* carrying the *firefly luciferase* gene and 0.3 μg/well pRL-TK plasmid carrying the *renilla luciferase* gene. Twenty-four hours after transfection, cells were lysed and luciferase activity was assayed using Dual-Luciferase Reporter Assay System kit according to manufacturer’s instructions (Promega Co., Madison, WI, USA). Relative luciferase activities are expressed as fold induction. All the experiments were performed in triplicates and the average is shown in each figure.

### 4.6. Human NSCLC Xenograft Models

For human NSCLC models, NSG (NOD-SCID-IL2Rgamma) mice were used. The NOD.Cg-Prkdc^scid^ Il2rg^tm1Wjl^/SzJ (Stock: 005557, NSG) mice were purchased from the Jackson Laboratory (Bar Harbor, ME USA) and bred in-house in pathogen-free conditions according to the protocols approved by the BRFAA Institutional Animal Care and Use Committee. Mice were kept under 12-h light:12-h dark conditions with free access to food and water. All food, cages, water, and other items that came in contact with mice were sterile.

Control and RelA/p65^KD^ A549 and H1437 cancer cells were subcutaneously inoculated into either side of immune-compromised 5-week-old male NSG (NOD-SCID-IL2Rgamma) mice (left side for WT and right side for KD cells) and were allowed to grow in vivo as tumour xenografts. Nine mice (*n* = 9) were studied per cell line. The mice were sacrificed at the end of 4 weeks for tumour weight measurement and for histopathologic and molecular analysis of the tumours [26]. Briefly, part of the tumours was fixed in 10% buffered formalin overnight, then transferred to 70% ethanol, and embedded in paraffin. Five-micrometre sections were cut and used for immunohistochemical analysis. A second part of the tumours (50–100 mg) was snap-frozen in liquid nitrogen and processed for molecular studies. All mice were housed in pathogen-free conditions according to the protocols approved by the BRFAA Institutional Animal Care and Use Committee.

### 4.7. RNA-Sequencing and Analysis

Unbiased RNA-Seq analysis was performed in three biological replicates per each cell line [26]. Briefly, total RNA quantity of the samples was measured with Nanodrop, while quality and integrity were verified with Agilent bioanalyser RNA 6000 nano kit. RNA-seq libraries were prepared with the Illumina TruSeq RNA v2 kit with 1 μg of total RNA input. QC of the RNA-Seq Libraries was performed with Agilent bioanalyser DNA1000 kit and quantification with Qubit HS. 75-bp single-end reads were generated with the Illumina NextSeq500 sequencer. RΝA-seq analysis was performed, using first of all the quality control program FastQC (https://www.bioinformatics.babraham.ac.uk/projects/fastqc/, accessed on 20 July 2018. Quality scores across all bases and across all samples were good. After checking the quality, we used the tool tophat2 with standard parameters for alignment. Hg19 reference genome was used. Samtools was then used to sort and filter the above, in order to get significant results. Last step to get the differential expression was to use cuffdiff with standard parameters. For GO analysis we used the online databases: David and EnrichR (https://david.ncifcrf.gov, accessed on 14 October 2020; https://maayanlab.cloud/Enrichr/, accessed on 19 October 2020). For GO analysis we used the online databases: David and EnrichR (https://david.ncifcrf.gov, https://maayanlab.cloud/Enrichr/, accessed on 19 October 2020). In R studio we utilised heatmap in order to produce the heatmaps. The Venn diagrams were produced from an online tool called meta-chart (https://www.meta-chart.com/venn, accessed on 31 July 2020).

### 4.8. Quantitative Real-Time PCR (qRT-PCR)

Total RNA from human lung cancer cell lines and from tumours grown in NSG mice was isolated using the TRI-Reagent (Merck, Kenilworth, NJ, USA), according to the manufacturer’s instructions. For cDNA synthesis, 0.5 μg of purified RNA was reverse-transcribed using the PrimeScript™ RT reagent kit (RR037A, Takara Bio Europe, Inc., Saint-Germain-en-Laye, France). For real time PCR analy-sis, 1 μL of cDNAs at 1:8 dilution cDNAs were amplified using KAPA SYBR^®^ FAST qPCR Master Mix (Kapa Biosystems, Inc., Wilmington, MA, USA) in a total volume of 10 μL according to the manufacturer’s instructions, in a Step One cycler (Applied Biosystems-ThermoFisher Scientific). Specific forward and reverse primers used included: CD82 forward 5′-GCTCATTCGAGACTACAACAGC-3′ and reverse 5′-GTGACCTCAGGGCGATTCA-3′, ROS1 forward 5′-TGTCTGCTGAATGAACCCCAA-3′ and reverse 5′-TGCCAGATCCCTGTGAATGAAA-3′, and RPII forward 5′-TCAATGCTGGTTTTGGTGACG-3′ and reverse 5′-GCATGTTGGACTCGATGCAG-3′. The relative fold change in gene expression was calculated with the 2(-ΔΔCT) method us-ing RPII as reference control. Cycle threshold (Ct) values ≥35 were considered as being background.

### 4.9. Isolation of Total Proteins and Western Blot Analysis

Cells were lysed in RIPA buffer as described previously [89,90]. Protein lysates of the mouse tumour xenografts were extracted by TRI-Reagent (Merck) according to the manufacturer’s instructions. Protein samples (40 μg) were analysed by SDS-PAGE followed by immunoblotting. Primary antibodies used were anti-NF-κΒ/p65 (sc-372), anti-phospho-NF-κΒ/p65 (Ser536) (sc-136548), anti-CD82 (sc-1087), anti-E-cadherin (sc-8426), anti-N-cadherin (sc-89987), anti-vimentin (sc-6260), anti-mCherry (AB0040-200, Abcam Co., Cambridge, UK), anti-p-ERK (sc-7383), anti-ERK (#9102, Cell Signalling Technology; CST, Danvers, MA, USA), anti-Akt1 (sc-5298), anti-Rac1 (#610650, BD Transduction Laboratories), anti-GAPDH (#2118, CST) and anti-β-actin (CloneAC15, A5441; Sigma-Aldrich Chemical Co., Milwaukee, WI, USA), followed by appropriate horseradish peroxidase (HRP)-conjugated secondary antibodies (Jackson Immunoresearch labs, UK). Immunoblots were developed using Clarity™ Western ECL Substrate (#170-5061, BioRad Labs, Inc., Hercules, CA, USA). The chemilluminescence signal was captured with a ChemiDoc XRS Molecular Imager and protein quantification was performed using the Quantity One 1-D Analysis Software version 4.6.9 (Bio-Rad Labs, Inc., Hercules, CA, USA).

For all panels of Western blots presented, the images of protein expression were copied and pasted on a white background to make composite blots. Brightness was slightly adjusted for uniformity and clarity similarly across all samples. However, there was no image manipulation. The panels in Figure 3F and in Figure 5D resulted from the joining of strips of different blots. For all quantification analysis raw, unprocessed images were used.

### 4.10. Indirect Immunofluorescence

Indirect immunofluorescence was performed as previously described [26,90]. The primary antibodies used were anti-CD82 (11-559-C100, Exbio Praha, AS, Vestec, Czech Republic) and anti-E-cadherin (sc-8426) followed by appropriate Alexa Fluor 488-conjugated secondary antibodies (Jackson ImmunoResearch labs, Cambridgeshire, UK). Cells were counterstained with DRAQ5 (Invitrogen-Thermo Fisher Scientific) for nuclei visualisation. The stained cells were washed with PBS and mounted on glass slides. Images were collected on a Leica TCS-SP scanning confocal microscope with 63× objective lens.

### 4.11. Tissue Sections and Immonohistochemistry

Tissue microarrays (TMA) were made as previously described [91]. In brief cylindrical cores were extracted from donor blocks and transferred to a receiver block with a TMA roboter (TMA grandmaster, 3D histech, Budapest, Hungary). TMAs contained 50 normal human lung tissue samples (*n* = 50), 50 samples from patients with LUAD (*n* = 50) and 50 samples from patients with LUSC (*n* = 50). The study was conducted according to the guidelines of the Declaration of Helsinki, and approved by the Institutional Review Board (or Ethics Committee). Informed consent of patients was given by all patients and the project was approved by the local scientific and ethics committees of the University Hospital of Ioannina, Greece (8/8-3-2019/Θ3) and the University Hospital of Heidelberg, Germany (#S315/20, 12 May 2020).

Whole tumour sections from consecutive archived LUAD (*n* = 16) and LUSC (*n* = 13) surgical specimens containing tumour and nearby lung parenchyma were used for immunohistochemical analysis. Initial diagnosis was bases on typical morphological features and TTF1/p63 immunohistochemical expression.

Protein expression of CD82, CD8 and Ki67 tissue specimens: An initial pre-treatment of the tissue sections involving deparaffinisation, rehydration and heat-induced epitope retrieval (HIER) was performed in a PT Link pre-treatment system (Dako-Agilent Technologies, Inc., Santa Clara, CA, USA) using the EnVision FLEX Target Retrieval Solution (#K8004, Dako-Agilent Technologies, Inc.) according to the manufacturer’s instructions. After pre-treatment, sections were immunostained on an Autostainer Link automated immunohistochemistry system (Dako-Agilent Technologies, Inc.) according to the manufacturer’s instructions. For CD82 protein expression, sections were incubated with anti-CD82 (11-559-C100, Exbio) at a 1:200 dilution for 20 min, anti-CD8 (M7103, Dako) at a 1:200 dilution for 20 min, or anti-Ki67 Clone MIB-1 (ZETA Corporation, Gunpo-city, Korea) at a 1:100 dilution for 30 min. After immunostaining sections were counterstained with haematoxylin. Finally, sections were dehydrated, cleared and mounted. Sections were observed under an Olympus BX41 optical microscope and digitally whole slide scanned with the Aperio AT2 Leica Biosystems scanner [92].

Immunohistochemical evaluation for CD8 T cells expression was performed as previously described [93] in a semiquantitative manner: 0: no cells, 1: few cells (<10%), 2: moderate number of positive cells (≥10% and <40%), and 3: abundant cells (≥40%), in the intra tumoural and peritumoural compartments. A binary system of low (scores 0 and 1) and high (scores 2 and 3) was used further for statistical purposed. CD82 expression was cytoplasmic and/or membranous and an at least 10% tumour cell expression was recorded as positive.

### 4.12. Wound Healing

For the wound healing assay 5 × 10^4^ cells were seeded in each well of a 24-well plate, in serum-free medium. After 24 h linear scratch wounds were made using a sterile pipette tip. Cells were washed with PBS to remove cell debris and incubated for another 24 h in serum-free medium. The wounds were observed under an optical microscope and photographed at the same position at 0 and 24 h with a Nikon camera. The percent of wound closure was calculated with the ImageJ software and the MRI (Montpellier Resources Imagerie) wound healing tool.

### 4.13. Bioinformatics Analysis

We validated CD82 in the Cancer Genome Atlas (TCGA) data sets, Gene Expression Profiling Interactive Analysis (GEPIA) database (http://gepia.cancer-pku.cn/, accessed on 27 February 2021, and UALCAN, a comprehensive and interactive web-resource for analysing cancer OMICS data (http://ualcan.path.uab.edu/cgi-bin/ualcan-res.pl, accessed on 27 February 2021). The pan-cancer cohort of TCGA was downloaded through cBioPortal (https://www.cbioportal.org/, accessed on 27 February 2021). Single cell analysis took place using the Single Cell Portal (https://singlecell.broadinstitute.org/single_cell, accessed on 27 February 2021).

### 4.14. Statistical Analysis

Numerical data are presented as mean of at least three independent experiments and bars represent ± SD. Statistical differences showed in graphs were calculated using Student’s *t* test or chi-square test as indicated in figure legends using the GraphPad Prism 5 software. *p*-values of <0.05 were considered statistically significant.

## 5. Conclusions

NF-κB RelA/p65 promotes lung epithelial cell tumour growth in vivo by downregulating the metastasis suppressor CD82 and enhancing the epithelial-to-mesenchymal cell transition via integrin-mediated signalling involving the mitogenic ERK, Akt1 and Rac1 proteins.

## Data Availability

Not applicable.

## Supplementary Materials

The following are available online at https://www.mdpi.com/article/10.3390/cancers13174302/s1: Figure S1. NF-κB luciferase reporter assays of A549 and H1437 cells. Figure S2. Growth of vector control and p65^KD^ A549 and H1437 cells. Figure S3. Expression of CD82 in LUAD and LUSC. Figure S4. Analysis of the immunohistochemical expression of CD8 in whole sections of early and advanced human LUAD and LUSC. Figure S5. Single cell analysis of human immune cells in lung cancer. Figure S6. Expression and localisation of E-cadherin protein in vector control and RelA/p65^KD^ H1437 human NSCLC cell line. Figure S7. Growth curves of vector control mCherry and mCherry-CD82^OE^ A549 and H1437 lung cancer cells. Figure S8. Generation of CD82^KD^ human NSCLC cells. Table S1. Clinicopathological variables and statistical analysis of the immunohistochemical staining of CD8, a marker of TILs, in FFPE whole tissue sections from NSCLC patients. Table S2. Bioinformatics analysis revealed a link between NF-κB RelA/p65, and integrin signalling pathways.
