# Supplementary material for: Canonical NF-κB Promotes Lung Epithelial Cell Tumour Growth by Downregulating the Metastasis Suppressor CD82 and Enhancing Epithelial-to-Mesenchymal Cell Transition
Source: Cancers (Basel). 2021 Aug 26;13(17):4302. doi: 10.3390/cancers13174302 (PMC8428346; doi:10.3390/cancers13174302)
Supplement: Supplementary file 1 [file cancers-13-04302-s001.zip › cancers-1312221-supplementary.pdf]

cancers

MDPI

Article

Canonical NF-κB Promotes Lung Epithelial Cell Tumour Growth by Downregulating the Metastasis Suppressor CD82 and Enhancing Epithelial-to-Mesenchymal Cell Transition

Eugenia Roupakia [1,2], Evangelia Chavdoula [2,3,†], Georgia Karpathiou [4,‡], Giannis Vatsellas [3], Dimitrios Chatzopoulos [3], Angeliki Mela [5], Jennifer M. Gillette [6], Katarina Kriegsmann [7], Mark Kriegsmann [8], Anna Batistatou [4], Anna Goussia [4], Kenneth B. Marcu [3,9], Emmanouil Karteris [10], Apostolos Klinakis [3] and Evangelos Kolettas [1,2,*]

Supplementary Materials

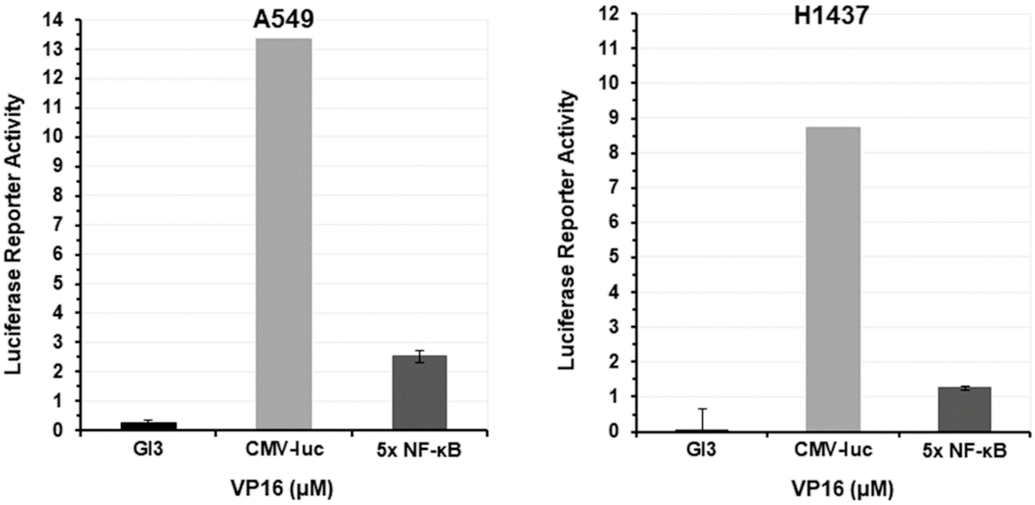

Figure S1. NF-κB luciferase reporter assays of A549 and H1437 cells.

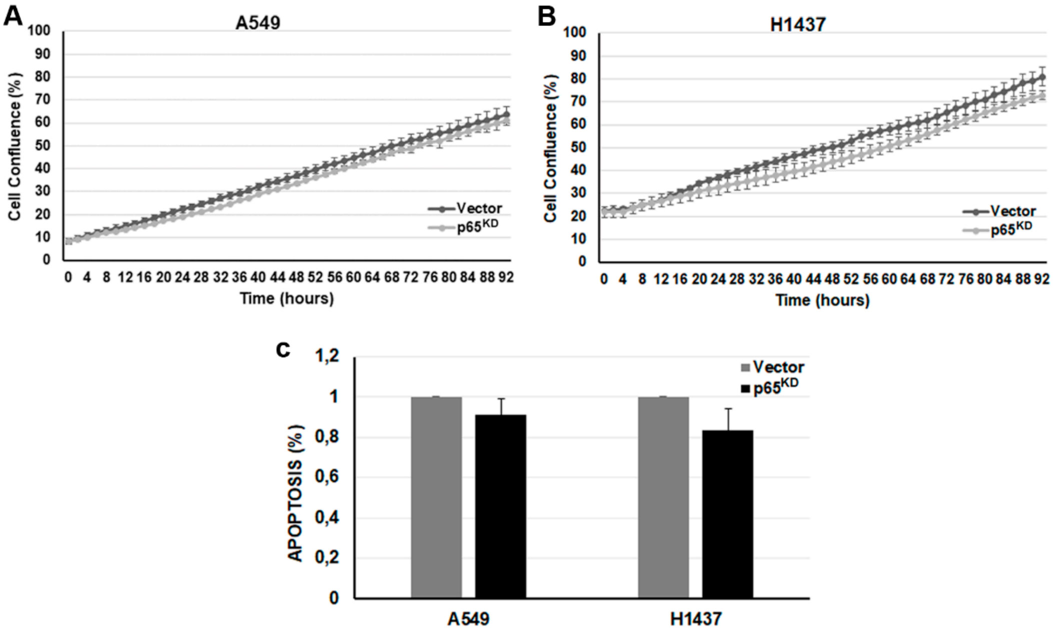

Figure S2. Growth of vector control and p65KD A549 and H1437 cells.

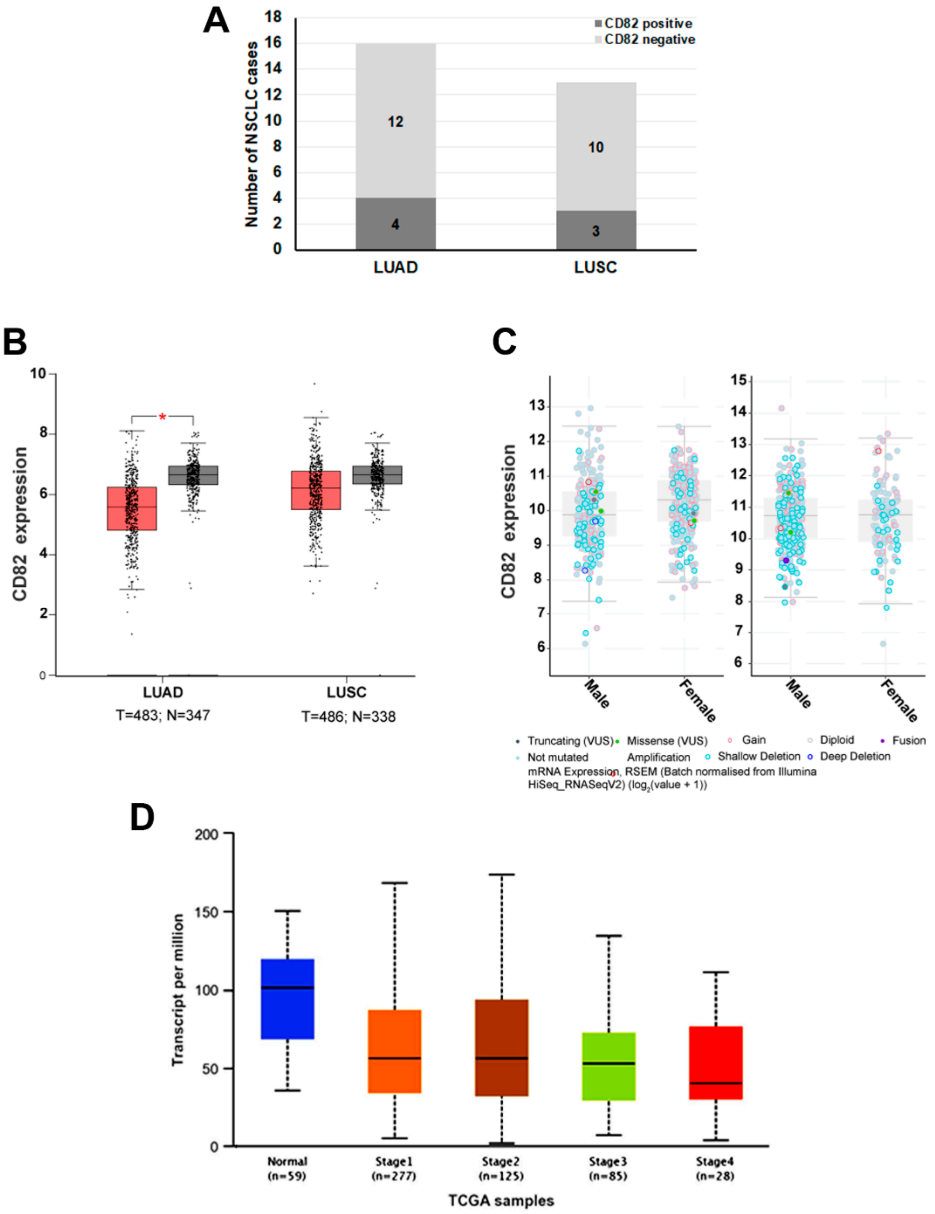

Figure S3. Expression of CD82 in LUAD and LUSC.

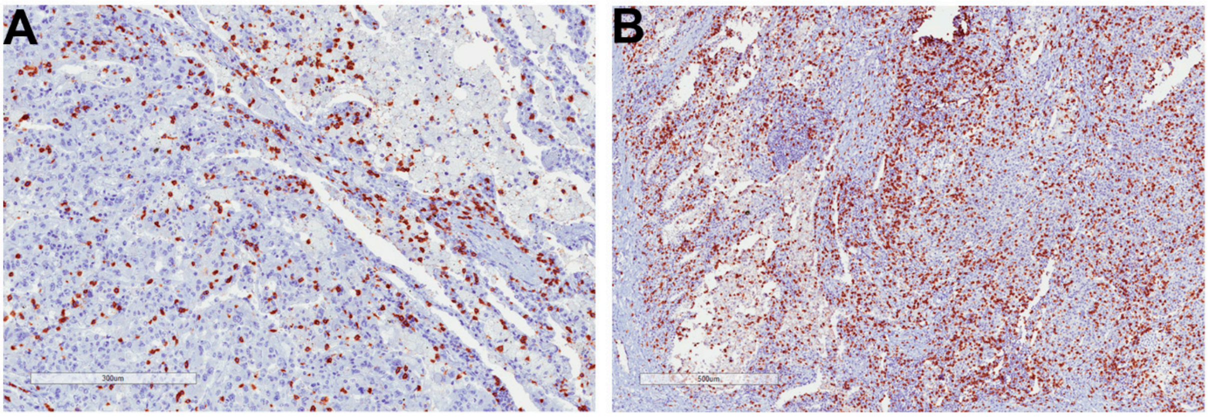

Figure S4. Analysis of the immunohistochemical expression of CD8 in whole sections of early and advanced human LUAD and LUSC.

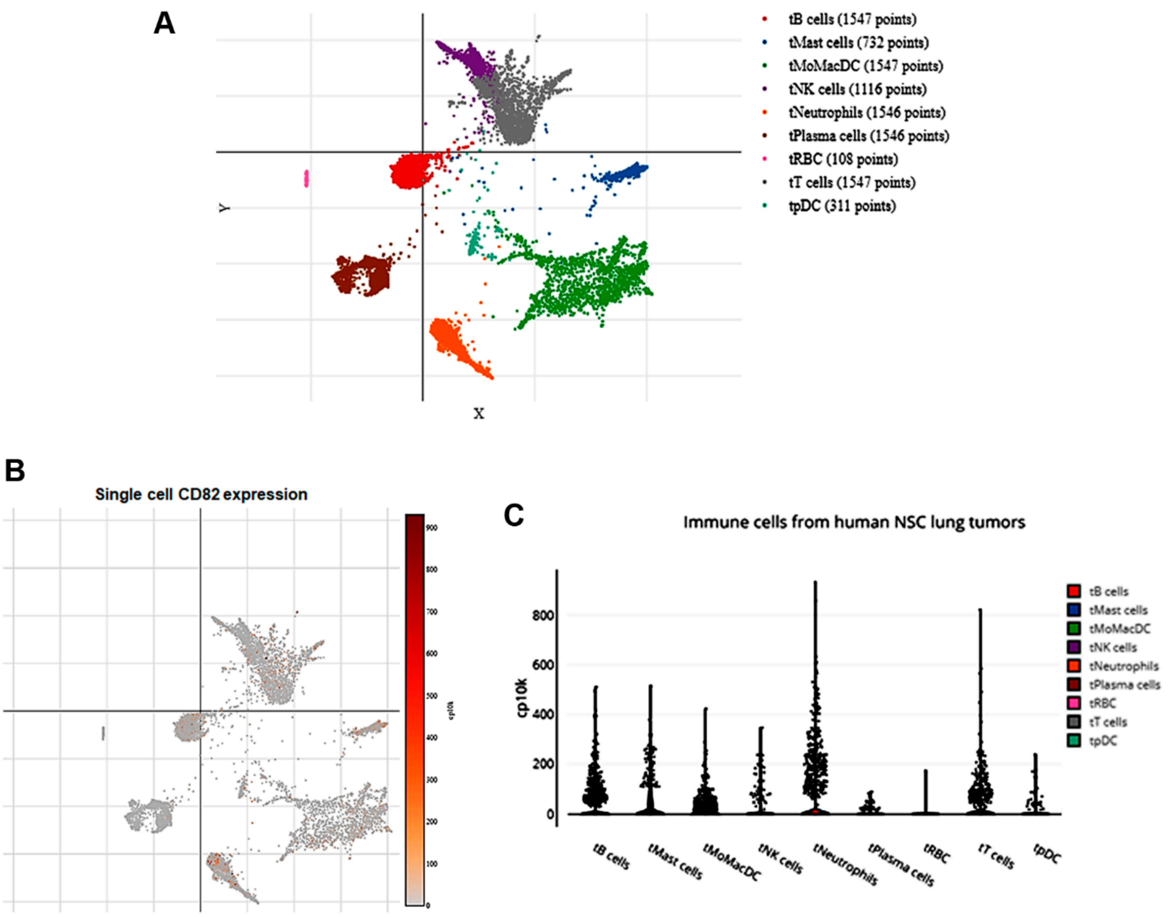

Figure S5. Single cell analysis of human immune cells in lung cancer.

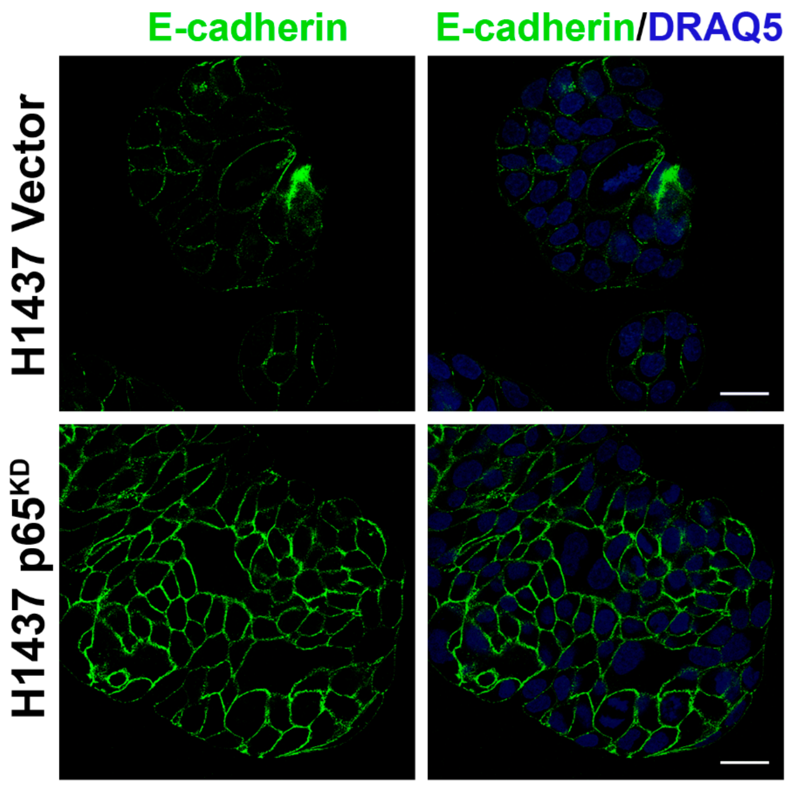

Figure S6. Expression and localisation of E-cadherin protein in vector control and RelA/p65KD H1437 human NSCLC cell line.

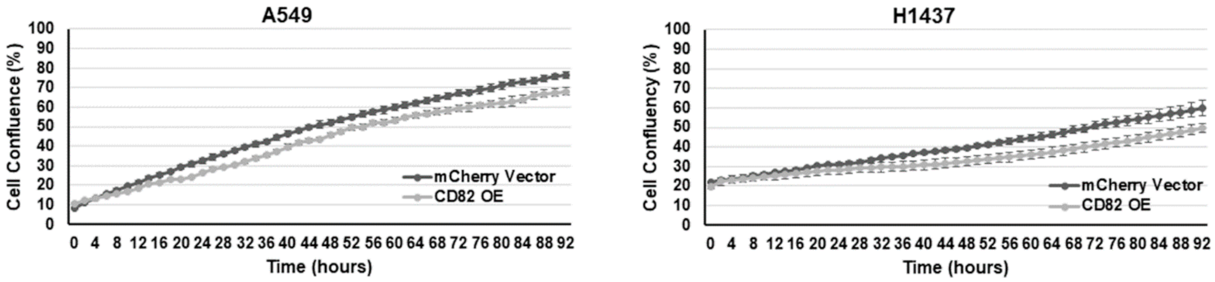

Figure S7. Growth curves of vector control mCherry and mCherry-CD82OE A549 and H1437 lung cancer cells.

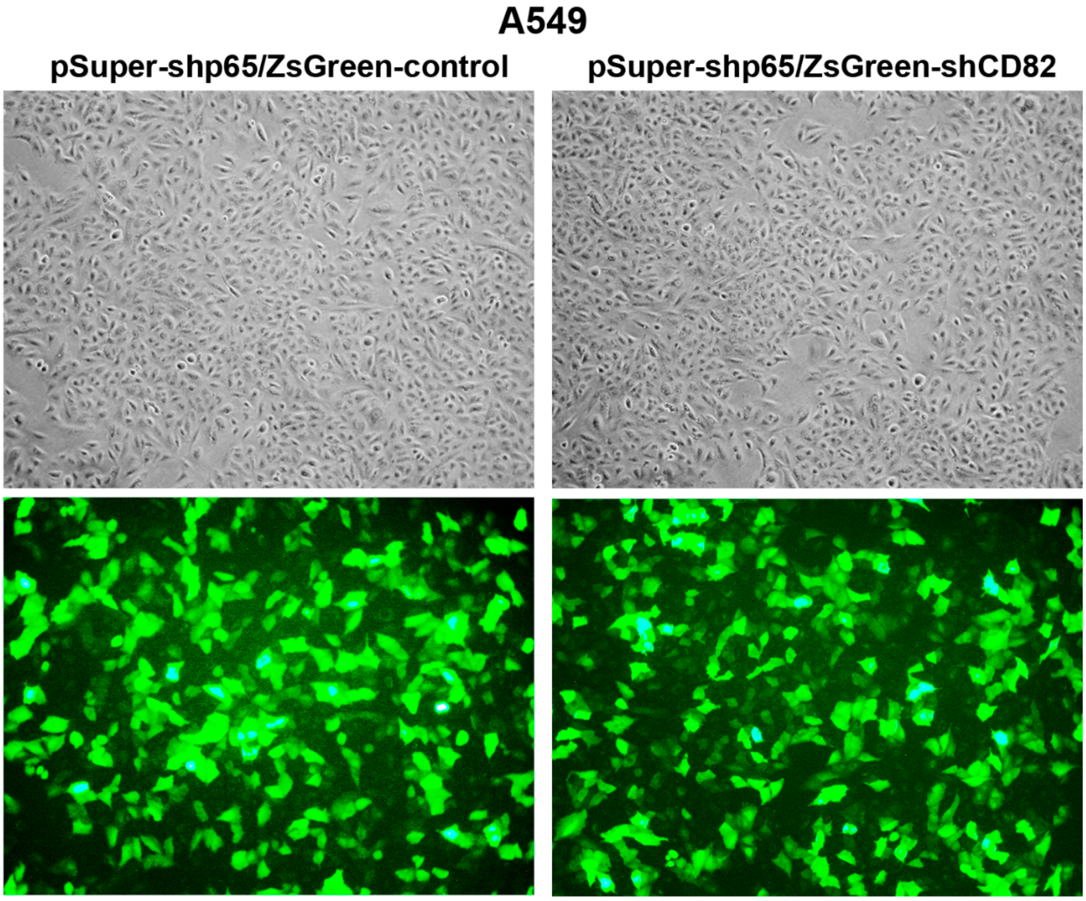

Figure S8. Generation of CD82KD human NSCLC cells.

Table S1. Clinicopathological variables and statistical analysis of the immunohistochemical staining of CD8, a marker of TILs, in FFPE whole tissue sections from NSCLC patients. Lung adenocarcinoma (LUAD) and squamous cell carcinoma (LUSC).

Clinico-pathological. variables	Number of cases
**Histological type**	
LUAD	16
LUSC	13
**Stage**	
Early	10
Advanced	19
**Age**	
<60	1
≥60	28
**Sex**	
Male	25
Female	4

Expression	CD8$^+$ intra-tumoural			CD8$^+$ peri-tumoural		
**CD82**	**0–1**	**2–3**	***p*–value**	**0–1**	**2–3**	***p*-value**
Positive	5	2	0.1	3	4	0.5
Negative	7	13		6	14	

Fisher’s exact test.Expression levels: 0 = No expression; 1 = Mild; 2 = Moderate; 4 = Intense

Table S2. Bioinformatics analysis revealed a link between NF- B RelA/p65, and integrin signalling pathways. Bioinformatics analysis using the Nature pathways showed that both cell lines, A549 and H1437, share commonly affected pathways such as integrin signalling pathways.

**A549**	
**Pathway (NCI-Nature 2016)**	***p*-value**
β3 integrin cell surface interactions	0.000002
β2 integrin cell surface interactions	0.000135
β1 integrin cell surface interactions	0.000345
Urokinase-type plasminogen activator (uPA) and uPAR-mediated signalling	0.000579
Alternative NF-κB pathway	0.001219
Ephrin B reverse signalling	0.002551
Osteopontin-mediated events	0.002805
IL23-mediated signalling events	0.004659
Atypical NF-κB pathway	0.010346
Canonical NF-κB pathway	0.018570
**H1437**	
**Pathway (NCI-Nature 2016)**	**p-value**
Validated transcriptional targets of AP1 family members Fra1 and Fra2	0.0007
α9β1 integrin signalling events	0.0043
β1 integrin cell surface interactions	0.0087
Signalling mediated by p38γ and p38δ	0.0110
Integrins in angiogenesis	0.0143
Syndecan-1-mediated signalling events	0.0156
EphrinB-EPHB pathway	0.0263
Integrin family cell surface interactions	0.0266
β5, β6, β7 and β8 integrin cell surface interactions	0.0374
SHP2 signalling	0.0402
